# Zero-fluoroscopy approach for ablation of supraventricular tachycardia using the Ensite NavX system: a multicenter experience

**DOI:** 10.1186/s12872-020-01344-0

**Published:** 2020-02-03

**Authors:** Guangzhi Chen, Yan Wang, Riccardo Proietti, Xunzhang Wang, Feifan Ouyang, Chang Sheng Ma, Rong Hui Yu, Chunxia Zhao, Kezhong Ma, Jie Qiu, Qigong Liu, Dao Wen Wang

**Affiliations:** 1 Division of Cardiology, Department of Internal Medicine, Tongji Hospital, Tongji Medical College, Huazhong University of Science and Technology, Wuhan, 430030 People’s Republic of China; 2 Department of Cardiac, Thoracic, and Vascular Sciences, via Giustiniani 2, 35121 Padua, Italy; 3 Heart Institute, Cedars Sinai Medical Center, Los Angeles, CA USA; 4 Asklepios Klinik St. Georg, Hamburg, Germany; 5 Department of Cardiology, Beijing Anzhen Hospital, Capital Medical University, Beijing, 100029 People’s Republic of China; 6 Department of Cardiology, Xiangyang Central Hospital, Xiangyang, 441021 People’s Republic of China

**Keywords:** Supraventricular tachycardia, Radiofrequency ablation, Zero-fluoroscopy, Radiation exposure

## Abstract

**Background:**

Three-dimensional electroanatomic mapping systems have demonstrated a significant reduction in radiation exposure during radiofrequency catheter ablation procedures. We aimed to investigate the safety, feasibility and efficacy of a completely zero-fluoroscopy approach for catheter ablation of supraventricular tachycardia using the Ensite NavX navigation system compared with a conventional fluoroscopy approach.

**Methods:**

A multicenter prospective non-randomized registry study was performed in seven centers from January 2013 to February 2018. Consecutive patients referred for catheter ablation of supraventricular tachycardia were assigned either to a completely zero-fluoroscopic approach (ZF) or conventional fluoroscopy approach (CF) according to the operator’s preference. Patients with atrial tachycardia were excluded.

**Results:**

Totally, 1020 patients were enrolled in ZF group; 2040 patients ablated by CF approach were selected for controls. There was no significant difference between the zero-fluoroscopy group and conventional fluoroscopy group as to procedure time (60.3 ± 20.3 vs. 59.7 ± 22.6 min, *P* = 0.90), immediate success rate of procedure (98.8% vs. 99.2%, *P* = 0.22), arrhythmia recurrence (0.4% vs. 0.5%, *P* = 0.85), total success rate of procedure (98.4% vs. 98.8%, *P* = 0.39) or complications (1.1% vs. 1.5%, *P* = 0.41). Compared with the conventional fluoroscopy approach, the zero-fluoroscopy approach provided similar outcomes without compromising the safety or efficacy of the procedure.

**Conclusion:**

The completely zero-fluoroscopy approach demonstrated safety and efficacy comparable to a conventional fluoroscopy approach for catheter ablation of supraventricular tachycardia, and mitigated radiation exposure to both patients and operators.

**Trial registration:**

clinicaltrials.gov Identifier: NCT03042078; first registered February 3, 2017; retrospectively registered.

## Background

Over the past two decades, the cardiac electrophysiology field has undergone great changes and development. Catheter ablation (CA) has become the gold standard for the treatment of symptomatic and recurrent supraventricular tachycardia (SVT). The success rate for this procedure is greater than 90% for supraventricular tachycardia such as atrioventricular nodal reentrant tachycardia (AVNRT) and atrioventricular re-entrant tachycardia (AVRT) [1]. Radiofrequency catheter ablation (RFCA) procedures are traditionally performed under the guidance of fluoroscopy that is a highly effective way to navigate catheters and to monitor their location [2]. However, fluoroscopy requires the administration of ionizing radiation, which carries non-negligible stochastic and deterministic effects on health for both patients and staffs [3]. These effects are cumulative and give rise to great concerns especially in a younger population, which highlights the importance of reducing radiation exposure during cardiac electrophysiology procedures [4]. Thus, the American College of Cardiology recommends the adoption of the “ALARA” (as low as reasonably achievable) principle in all interventional laboratories [5].

In recent years, non-fluoroscopic three-dimensional electroanatomic mapping systems (such as Ensite NavX and Carto) have been introduced to facilitate catheter ablation procedures [6]. Some studies have demonstrated these systems enable ablation of supraventricular tachycardia (SVT) substrates with significantly decreased radiation exposure to both patients and staffs compared to conventional fluoroscopy-guided ablation [7–10]. However, there are only a few experiences published of using three-dimensional electroanatomic mapping systems with a completely zero-fluoroscopy (ZF) approach.

Therefore, the aim of the present study was to explore the safety, feasibility, and efficacy of a completely zero-fluoroscopy (ZF) approach for catheter ablation of supraventricular tachycardia (SVT) guided by the Ensite NavX system compared with a conventional fluoroscopy (CF) approach.

## Methods

### Study design

This prospective non-randomized registry study was performed in seven geographically diverse Chinese arrhythmia centers. It was approved by the institutional ethical review board of Tongji hospital, Tongji Medical College, Huazhong University of Science and Technology according to the guidelines for good clinical practice and the Declaration of Helsinki.

Between January 2013 and February 2018, consecutive patients with supraventricular tachycardia, including AVNRT and AVRT, were enrolled in this study. The patients with atrial tachycardia were excluded. Routinely, except manifest pre-excitation syndrome, all patients with supraventricular tachycardia were arranged to undergo transesophageal electrophysiological study (TEEPS) before the procedure. Eligible participants were assigned to either the ZF or CF group according to the operator’s preference. Since more operators utilized conventional approach, the sample ratio of ZF to CF was about 1:2.

The ZF approach was performed using Ensite NavX system as the only guidance (St Jude Medical, St. Paul, MN, USA) and the operating staffs did not wear lead protection. The CF approach utilized fluoroscopy imaging with or without three-dimensional mapping system.

Cardiac magnetic resonance imaging, computed tomography, and intracardiac echocardiography were not used during the procedure. Independent operators working in seven centers participated in this study.

Written informed consents were obtained from all patients before the procedure. All the patients underwent routine blood biochemistry, electrocardiography, chest X-ray imaging, and cardiac echocardiography before the procedure. Antiarrhythmic drugs were discontinued for a minimum of five half-lives prior to the procedure.

### Operative procedures

All procedures were performed under local anesthesia and without sedation.

### Zero-fluoroscopy (ZF) approach

**Fig. 1:**
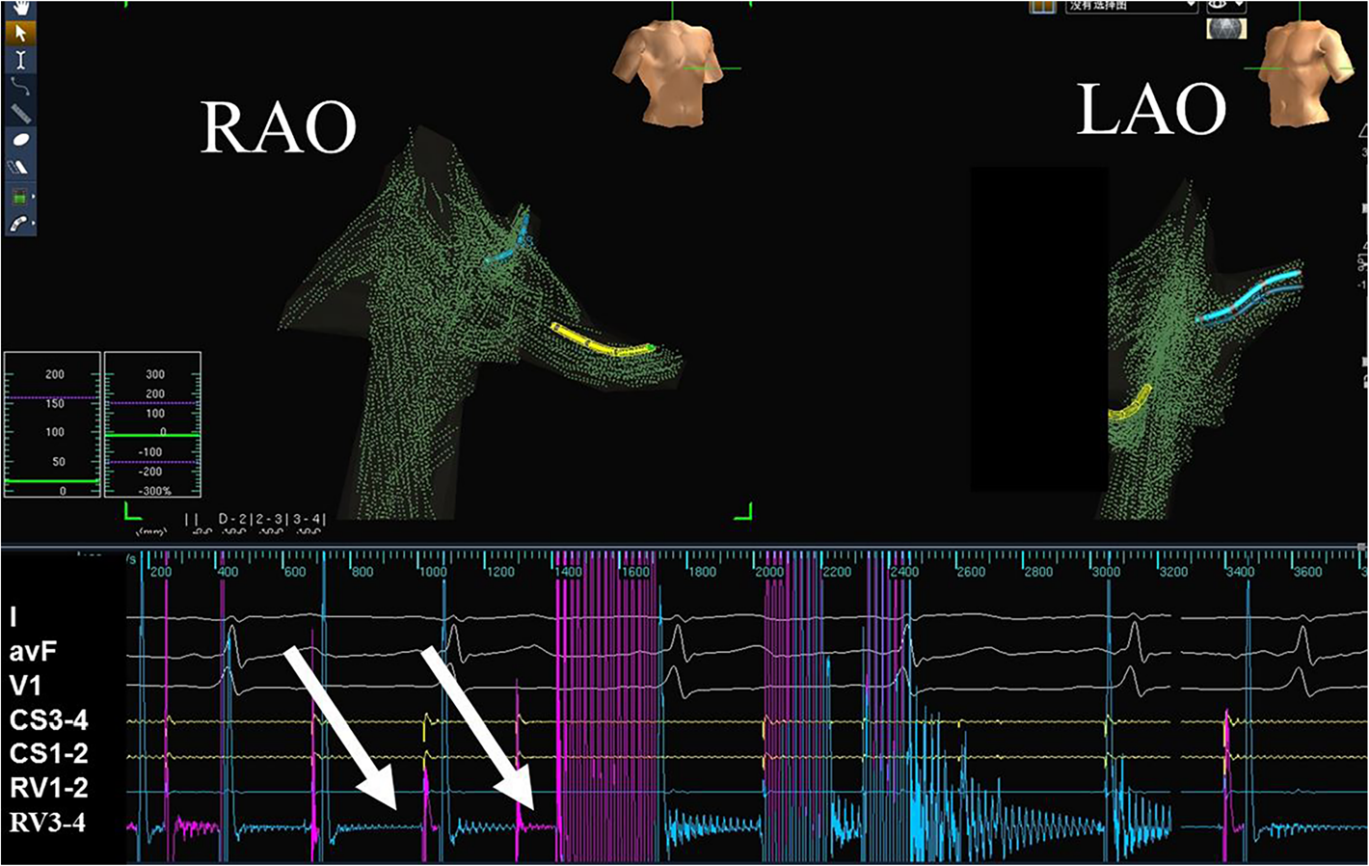
A J-shaped wire was placed into a subclavian vein. A correct insertion was verified by the characteristic interference signal, which was produced by rotating the J-shaped wire when the first catheter had been positioned at the middle of right atrium. The white arrows indicate the vertical magenta line associated with the interference signal. Abbreviations: LAO, left anterior oblique view; RAO, right anterior oblique view

**Fig. 2:**
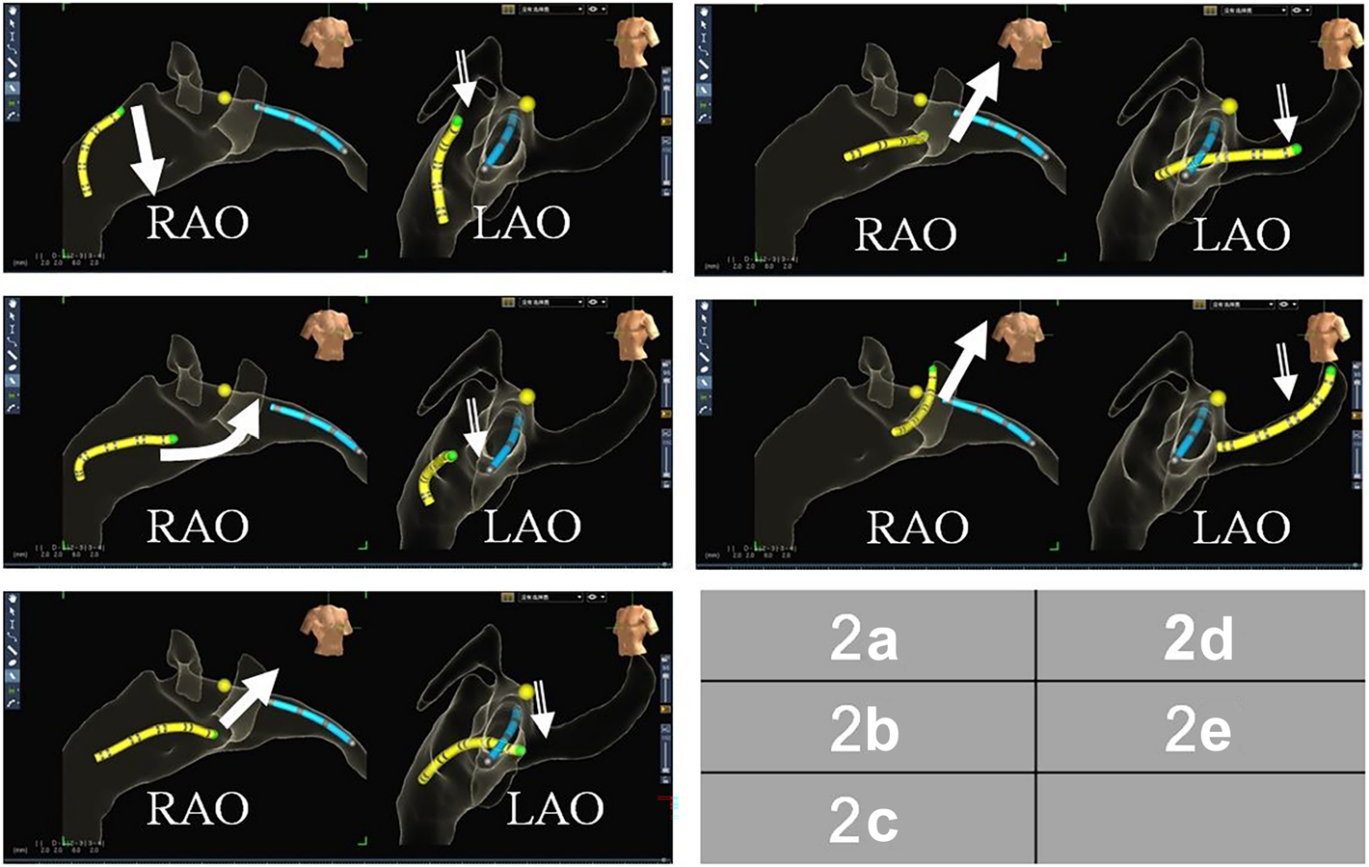
Stepwise approach to placement of the steerable-curve electrode into a coronary sinus via the femoral vein. Generally, a fixed-curve quadripolar electrode was placed into the apex of right ventricle (as shown in blue bands); then His potential was recorded by the electrode and marked by yellow dot. **a** First, the steerable electrode was advanced to the position about one centimeter below the position of his potential or peak of tricuspid valve. **b** Secondly, it was flexed toward the right ventricle. **c** Thirdly, it was rotated backward to locate the orifice of coronary sinus. **d** Fourthly, the electrode was extended gently. **e** Finally, the tip of electrode was advanced distally into coronary sinus. The solid arrow and blank arrow showed the track of catheter tip in RAO and LAO, respectively

The Ensite NavX system was used for catheter positioning and mapping. Catheters were generally first inserted into the heart through the femoral veins in biplane views that were used to visualize the path of the catheters. Two quadripolar catheters were introduced via the femoral vein and positioned in the right ventricular apex and at the His bundle, respectively. A steerable decapolar electrode was then placed in the coronary sinus (CS) via the femoral vein. Once the catheter was inserted into the CS, system optimization and respiratory compensation were performed. A general right atrial geometry was constructed from the placed catheters during positioning and the electrophysiology study was routinely performed in accordance with standard protocols (Figs. 1 and 2).

**Fig. 3:**
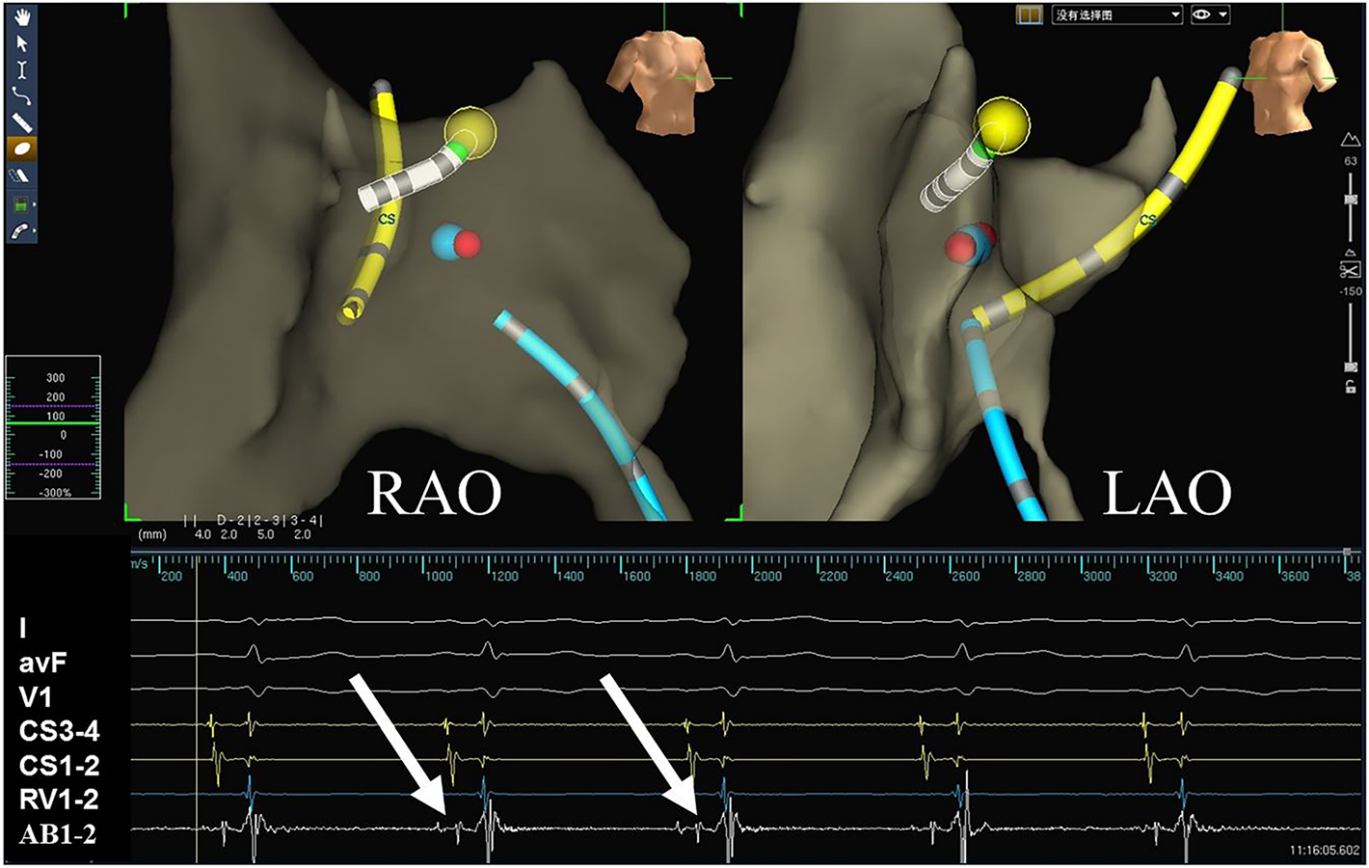
The virtual geometry of the targeted area in the right atrium was reconstructed during ablation of AVNRT. Upper panel: the ablation catheter was placed at the His bundle with the tip of the catheter shown in green, and the area of the His bundle is labeled with yellow dots, and the yellow and blue bands show the electrodes placed in the coronary sinus and in the right ventricle, respectively; typical slow pathway potential was mapped and marked with a blue dot and finally power delivery at 35 watts , which was marked as red dot, led to slow junctional beats. Lower panel: the white arrows show the His bundle electrogram recorded by the ablation catheter. AVNRT, atrioventricular nodal reentrant tachycardia; the abbreviations are as in Fig. 1

**Fig. 4:**
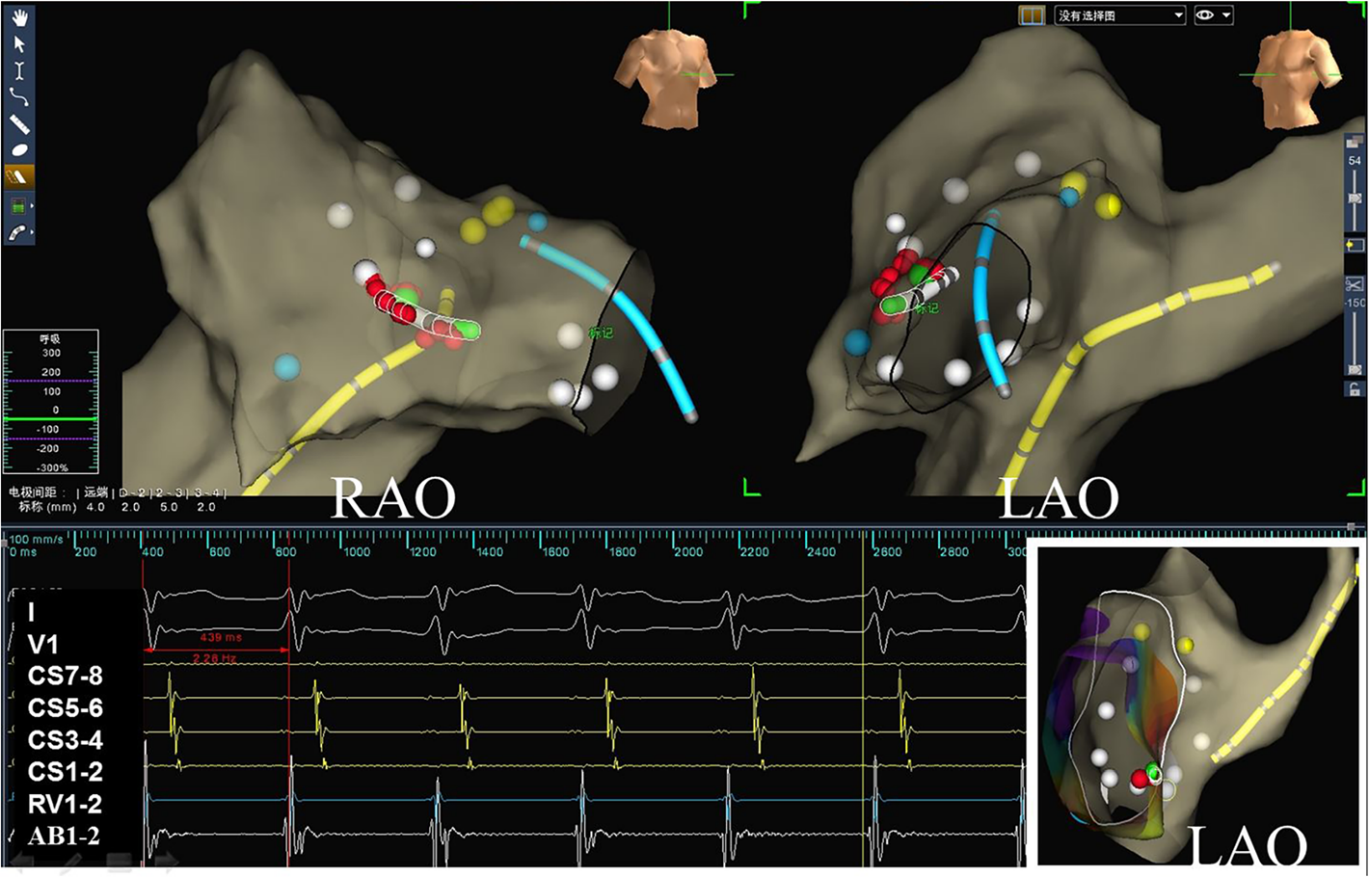
A patient with two manifest right sided accessory pathways was ablated using the ZF approach. Upper panel showed that the tricuspid valve was labeled with white dots and the site of the His bundle was labeled with yellow dots in RAO and LAO. The ablation catheter was placed at the first accessory pathway at about nine o’clock during tachycardia. Left lower panel showed the electrogram recording at the first target site. Right lower panel showed the second accessory pathway at 5 o’clock marked with red dot. Other abbreviations are as in Fig. 1

**Fig. 5:**
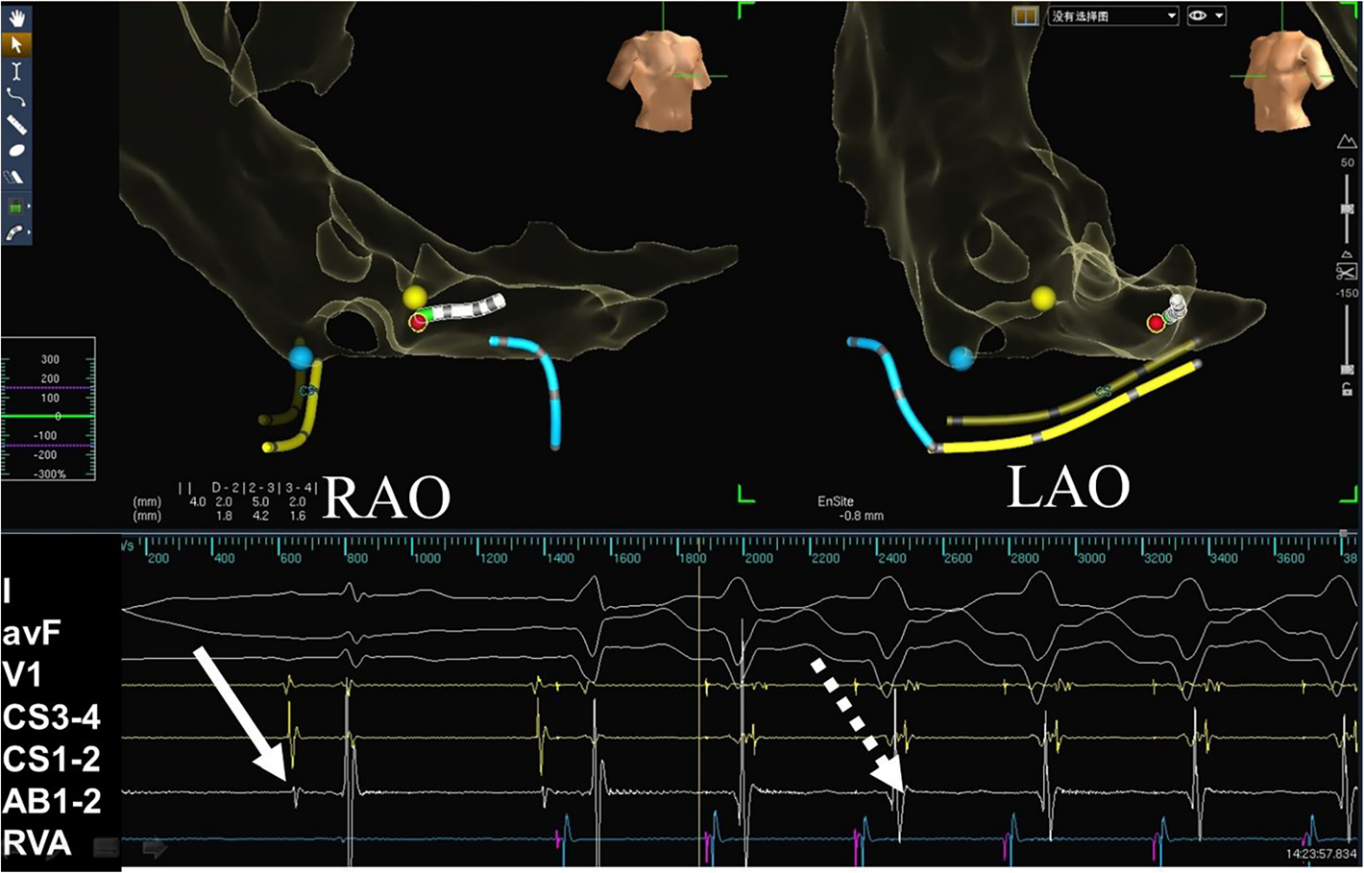
A concealed left accessory pathway was mapped and ablated using the ZF approach. The two white arrows in lower panel shows the endocardial electrogram recorded by the ablation catheter (green tip) placed in the targeted ablation area through aorta via retrograde access. The bottom of noncoronary aortic cusp was marked with a blue dot and His potential was marked with a yellow dot. The solid white arrow marks the small “A” wave during sinus rhythm. The dashed white arrow indicates the earliest “A” wave fused in the “V” wave during right ventricular pacing. Abbreviations are as in Fig. 1

Standard protocols and procedures, depending on the arrhythmic substrate, were used for all ablation procedures [11]. For arrhythmias originating in a right heart chamber, the ablation catheter was introduced via a femoral vein. The mapping points were as follows: (1) for AVNRT, the slow pathway was ablated (Fig. 3); (2) for a right accessory pathway, the tricuspid valve was labeled using five to six white points according to electrophysiological characteristics before the ablation (Fig. 4). For left accessory pathways, the operator selected a retrograde aortic approach via the femoral artery to obtain access to the left side of heart. The positions of the aortic root and left sided His bundle were labeled using one or two blue or yellow points, respectively, and the accessory pathway was tagged along the electrode placed in the coronary sinus or other regions of the mitral annulus (Fig. 5).

### Conventional fluoroscopy (CF) approach

After local anesthesia, diagnostic electrode catheters were introduced via the femoral, right internal jugular, or subclavian veins; and were positioned in the high right atrium, right ventricle, bundle of His, and coronary sinus. The ablation catheter was introduced to the heart via the femoral vein. Ventricular and atrial stimulation protocols were used to assess conduction properties and arrhythmia inducibility. Standard protocols and procedures, depending on the arrhythmic substrate, were used for all ablation procedures [12]. Fluoroscopy was used throughout all phases of the procedure, including confirmation of the guidewire position, electrophysiological studies, mapping, and ablation.

### Data collection

All preoperative, operative, and follow-up data were collected and stored in excel spreadsheets by independent researchers and other independent researchers made follow-up.

The following data were collected on each patient: clinical and demographic variables (age, sex, body weight, height, arrhythmia type); procedure-related variables (procedure date, assigned group, ablation method and route, procedure time, fluoroscopy time, number of lesions, total ablation time, immediate success rate, complications, and recurrences during follow-up).

Procedure time (in minutes) was defined as the interval from the beginning of local anesthesia to extraction of all femoral venous sheaths at the end of the procedure. Fluoroscopy time (in minutes) was defined as the total duration of exposure during the procedure. Total ablation time was calculated in seconds but time for tentative ablation was not taken into account. Number of lesions was defined as the number of ablation sites and tentative ablation was excluded. Routinely, tentative ablation would be terminated after 5 to 10 s of power delivery when pathway was not blocked. Procedural success for AVNRT was defined as the absence of inducible tachycardia either under basal condition or under isoproterenol stimulation. Procedural success for AVRT was defined as the non-inducibility of tachycardia, loss of pre-excitation (if manifest), loss of retrograde accessory pathway conduction and transient atrioventricular block induced by intravenous adenosine. Complications were defined as pseudoaneurysm, arterial-venous fistula, pneumothorax, second- or third-degree atrioventricular block, cardiac tamponade, or other serious complications requiring intervention.

### Follow-up

An independent technician performed follow-up at 1 month, 3 month, and 12 months after the ablation procedure. Generally, all the patients underwent physical examination and echocardiography at one month, and ECG each time during the follow-up. Any symptoms or signs related to complications or recurrence was recorded. An ECG and electrophysiology study would be performed to rule out recurrence when the patients had suspicious symptoms or signs.

### Statistical analysis

Data are shown as the mean ± standard deviation, whereas categorical data are expressed as numbers and percentages. Student’s t-tests, one-way analysis of variance, Chi-square tests, and Fisher’s exact tests were used to compare differences among groups. All analyses were performed using Statistical Package for the Social Sciences (SPSS) version 13.0 (IBM Inc., Armonk, NY, U.S.A.). All *P* values < 0.05 were considered statistically significant.

## Results

### Patient characteristics

Finally, 1020 cases were enrolled for ablation using ZF approach; 2040 consecutive cases ablated by CF approach were selected as control. Distributions of age, gender, weight, height, and the number of redo cases had no significance between the two groups. Both ZF and CF groups were well balanced with regard to demographics, clinical baseline characteristics, and the types of arrhythmia (Tables 1 and 2). The proportion of AVNRT and special, multiple, septal, right free wall and left free wall accessory pathway, and accessory pathway plus AVNRT were comparable and had no significance between ZF group and CF group (Table 2).

**Table 1:** Baseline characteristics of the enrolled patients in all groups

	ZF (*n* = 1020)	CF (*n* = 2040)	Total cohort (*n* = 3060)
Mean age (years)	45.4 ± 15.4	45.2 ± 15.3	45.3 ± 15.4
Weight (kg)	62.7 ± 11.8	64.5 ± 11.6	63.8 ± 11.7
Height (cm)	163.6 ± 8.4	165.9 ± 8.2	164.9 ± 8.3
Male patients	459 (45.0%)	908 (44.5%)	1367 (44.7%)
With AT/AF	4 (0.4%)	7 (0.3%)	11 (0.4%)
EPS Only	15 (1.5%)	35 (1.7%)	50 (1.6%)
SVT	1001 (98.1%)	1998 (97.9%)	2999 (98.0%)
Give up^a^	3 (0.3%)	5 (0.2%)	8 (0.3%)
Ablation	998 (97.8%)	1993 (97.7%)	2991 (97.7%)
Redo cases	5 (0.5%)	12 (0.6%)	17 (0.6%)

*Abbreviations: AT* atrial tachycardia, *AF* atrial flutter, *EPS* electrophysiological study, *ZF* zero-fluoroscopy, *CF* conventional fluoroscopy, *SVT* supraventricular tachycardia

^a^some patients refused to receive ablation owing to the possible risk after electrophysiology study

**Table 2:** Classification of supraventricular tachycardia in study patients

	ZF (*n* = 1020)	CF (*n* = 2040)	Total cohort (*n* = 3060)
SVT	1001 (98.1%)	1998 (97.9%)	2999 (98.0%)
1.AVNRT	556 (54.5%)	1052 (51.6%)	1608 (52.5%)
2.AP			
1) Septal	75 (7.4%)	160 (7.8%)	235 (7.7%)
2) Left free wall	261 (25.6%)	568 (27.8%)	829 (27.1%)
3) Right free wall	88 (8.6%)	173 (8.5%)	261 (8.5%)
4) Multiple	16 (1.6%)	31 (1.5%)	47 (1.5%)
3.AP + AVNRT	5 (0.5%)	14 (0.7%)	19 (0.6%)
(Special AP)			
(Para His)	6 (0.6%)	15 (0.7%)	21 (0.7%)
(Venous)	2 (0.2%)	5 (0.2%)	7 (0.2%)

*Abbreviations: AP* accessory pathway, *AVNRT* atrial ventricular node reentrant tachycardia, *AVRT* atrial ventricular reentrant tachycardia

As shown in Tables 1 and 2, the baseline characteristics, the types of arrhythmias, and the rate of redo cases in CF group were well balanced with those in ZF group. The mean follow-up period was 9.7 ± 4.0 months.

### Procedural features

#### Fluoroscopy use

##### ZF group

Among the 1020 cases enrolled in ZF group: (1) there were 1015 cases (99.5%) who completed electrophysiology study without fluoroscopy; (2) four cases (0.4%) were excluded because of atrial tachycardia or atrial flutter; (3) fifteen cases (1.5%) could not identify ablation target and only underwent electrophysiology study; (3) three young patients (0.3%) with para-His pathway finally refused to take ablation after electrophysiology study because the risk of atrioventricular block; (4) finally, 998 cases (97.8%) were planned for further ablation (Table 1).

Among the 998 cases planned for ablation, 7 cases (0.7%) switched to CF group because of the need of angiography; five of them were due to severe tortuous vessels; two of them were due to pathway suspected to be originated from diverticulum, although one of them actually was originated from the anterior margin of coronary sinus orifice. There were 99.3% (991/998) of patients who completed ablation without fluoroscopy (Table 3).

**Table 3:** Ablation outcome of the zero-fluoroscopy (ZF) approach compared with those of the conventional fluoroscopy (CF) approach

	ZF (*n* = 998)	CF (*n* = 1993)	*p*-value
Number of lesions^a^	3.6 ± 2.9	4.5 ± 3.5	< 0.01
Ablation time (seconds)	274.4 ± 207.1	301.5 ± 247.5	0.13
Fluoroscopy time (minutes)	0	6.9 ± 5.9	< 0.01
Procedure time (minutes)	60.3 ± 20.3	59.7 ± 22.6	0.90
Switch to CF (*n*, %)	7 (0.7%)	NA	NA
Complete ZF (*n*, %)	991 (99.3%)	NA	NA
Immediate failure (*n*, %)	5 (0.5%)	15 (0.8%)	0.43
Immediate success (*n*, %)	986 (98.8%)	1978 (99.2%)	0.22
Recurrence (*n*, %)	4 (0.4%)	9 (0.5%)	0.85
Total success (*n*, %)	982 (98.4%)	1969 (98.8%)	0.39

*Abbreviations: NA* not applicable; other abbreviations were seen as in Table 1

^a^tentative ablations of less than 10 s were not included

##### CF group

All the procedures in CF group were performed fluoroscopy. The mean total fluoroscopy time was 6.9 ± 5.9 min in the CF group (Table 3).

#### Number of ablated lesions

The ZF approach required fewer lesions for ablation than did the CF approach (3.6 ± 2.9 vs. 4.5 ± 3.5; *P* < 0.01) (Table 3).

#### Procedure time

As shown in Table 3, the procedure time of ZF and CF group was 60.3 ± 20.3 and 59.7 ± 22.6 min respectively (Table 3). The results were similar and had no significance between two groups (*P* = 0.90).

#### Success and recurrence rates

Immediate ablation success was achieved in 986 (98.8%) of the ZF approach patients and in 1978 (99.2%) of the CF approach patients (Table 3). The ZF and CF approaches had similar rates of total success (98.4% vs. 98.8%, *P* = 0.39) and arrhythmia recurrence (0.4% vs. 0.5%, *P* = 0.85) (Table 3).

#### Complications

Over the course of the study a total of forty complications were observed. The total complication rates were similar in the two groups (1.1% vs. 1.5%, *P* = 0.41). In the ZF group, there were six pseudoaneurysms, four arterial-venous fistulas, and one case of 2nd or 3rd degree atrioventricular block. In the CF group, there were fourteen pseudoaneurysms, six arterial-venous fistulas, six cases with pneumothorax, two cases of 2nd or 3rd degree atrioventricular block, and one cardiac tamponade. There were no serious complications that required thoracic surgery. The occurrence of pneumothorax was higher in CF group compared with the ZF group (0.3% vs. 0.0%, *P* = 0.01). The higher incidence of pneumothorax in CF group might be due to more cases of subclavian access whereas almost all the cases in ZF group just use femoral access. No specific complications were related to zero-fluoroscopy technique (Table 4).

**Table 4:** Complications in zero-fluoroscopy (ZF) and conventional fluoroscopy (CF) groups

Complications	ZF (*n* = 998)	CF (*n* = 1993)	*p*-value
Pseudoaneurysm, *n*	6 (0.6%)	14 (0.7%)	0.75
Arterial-venous fistula, *n*	4 (0.4%)	6 (0.3%)	0.66
Pneumothorax, *n*	0 (0.0%)	6 (0.3%)	0.01
II-III degree of AVB, *n*	1 (0.1%)	2 (0.1%)	0.99
Cardiac tamponade, *n*	0 (0.0%)	1 (0.1%)	0.32
Thoracic surgery, *n*	0 (0.0%)	0 (0.0%)	NA
Total (*n*, %)	11 (1.1%)	29 (1.5%)	0.41

*Abbreviations: AVB* atrial ventricular block, *NA* not applicable; other abbreviations were seen as in Table 1

### The learning curve of the ZF approach

**Fig. 6:**
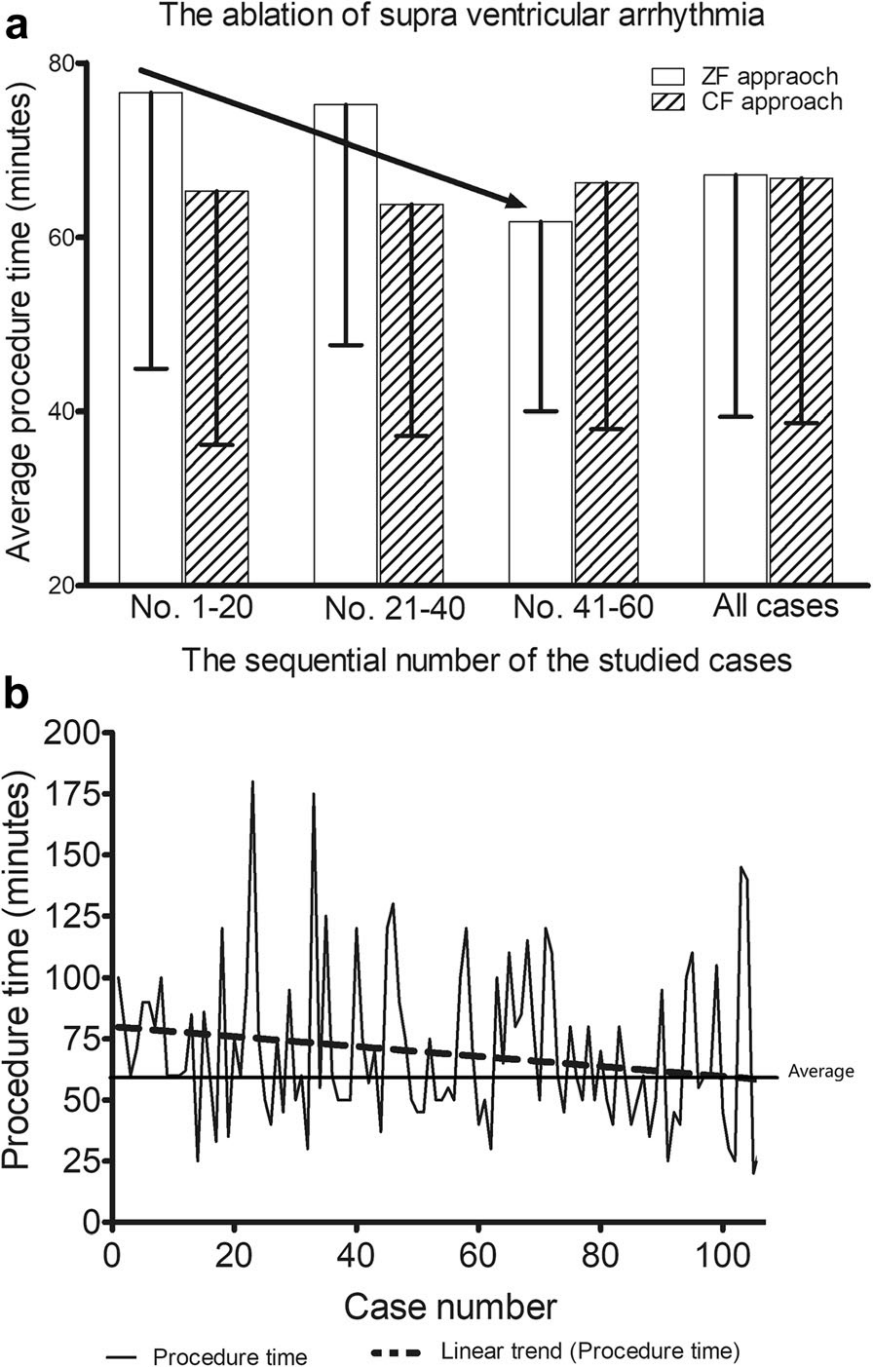
**a** The learning curve of the zero-fluoroscopy (ZF) approach during the ablation of supraventricular arrhythmia. Panel showed the average procedure time for the 1st to 20th cases, 21st to 40th cases, 41st to 60th cases, and all cases when each of the two approaches was used. **b** Graph showed linear trend of the procedure time for the first 120 ablations performed using zero-fluoroscopy approach

Figure 6a showed the average procedure time of the two approaches for the ablation of supraventricular arrhythmias according to procedure quartile, 1st to 20th, 21st to 40th, and 41st to 60th cases, and that of all cases. The average procedure time of the ZF approach was about 10 min longer than that of the CF approach during the ablation of 1st to 20th, 21st to 40th cases. No statistically significant reduction in average procedure time was observed between ZF approach group and CF approach group.

Figure 6b showed linear trend of the procedure time for the first 120 ablations performed using zero-fluoroscopy approach. The procedure time showed a gradually reduction trend and nearly reduced to average procedure time about after 60 to 80 cases of operation.

## Discussion

The present study investigated the safety, efficiency, and efficacy of a zero-fluoroscopy approach compared with a conventional fluoroscopy approach. The zero-fluoroscopy approach used the Ensite NavX system as the sole imaging modality for guiding procedures during the ablation of supraventricular tachyarrhythmia including AVNRT and AVRT. Our results demonstrated that the zero-fluoroscopy approach had equivalent safety, efficiency, and efficacy as the conventional approach, which utilized fluoroscopy with or without three dimensional mapping systems [13].

As we all know, fluoroscopic radiation is harmful to both medical staff and patients, particularly in children, pregnant women, and some specific adults. Various efforts and measures have been explored for reducing radiation exposure including adjustments in fluoroscopic technique, the use of lead protective garments, and the introduction of additional imaging technologies, such as electro-anatomical mapping or ultrasound [14]. Optimal settings of fluoroscopic parameters, such as pulse rate, collimation, detector sensitivity/distance, and signal filtration can significantly diminish radiation exposure [15]. Medical staffs who wear lead garments also reduce exposure to ionizing radiation; however, heavy lead apparel increases workplace discomfort and the risk of neck, back, and joint complications. Some studies have confirmed that interventional cardiologists had experienced an alarming incidence of spinal complaints [16]. Intracardiac echocardiography (ICE) has allowed identification of anatomic structures and potential anatomic variations, and has served to guide catheter navigation, which minimized radiation exposure to operators and patients [17]. Moreover, intracardiac echocardiography not only increases procedural expense but also requires additional venous sheath insertion, which might be an issue in younger patients [18]. The development of three dimensional electroanatomic mapping systems has allowed interventional electrophysiologists to significantly reduce the amount of radiation exposure to both staff and patients [19].

Recently, the Ensite NavX system has been investigated for the near-complete or complete elimination of radiation exposure during SVT ablation procedures [20]. Fernández-Gómez et al. reported the application of the Ensite NavX system as a sole imaging method for guiding procedures, allowing complete elimination of fluoroscopy in 94.7% of right-sided SVT cases [21]. Stec et al. documented that a minimally invasive and simplified approach with the Ensite NavX system navigation resulted in complete elimination of fluoroscopy and use of protective lead aprons by the electrophysiology staff in approximately 95% of SVT ablations, without changing the acute, long-term success or adverse event rates [22]. Results of a prospective, randomized, single-center study from Ma et al. demonstrated that the Ensite NavX system navigation approach significantly reduced fluoroscopy exposure during right-sided accessory pathway ablation in adults [23]. The NO-PARTY multicenter randomized controlled trial compared conventional fluoroscopy-guided procedures with procedures performed using Ensite NavX system as the primary catheter visualization tool in patients undergoing electrophysiology study for supraventricular tachycardias; the results showed that a minimally fluoroscopic approach in the ablation of SVTs dramatically reduced patients’ exposure, risks of cancer incidence and mortality, and years of life affected and lost, while maintaining safety and efficacy [10].

Gist K et al. demonstrated that procedure time significantly shortened as a function of experience. After an adequate learning curve, the procedure can be performed in a very acceptable amount of time [24]. To the best of our knowledge, previous studies have not made a comparison with conventional method in a large patient cohort. This study compared the zero-fluoroscopy approach with a conventional fluoroscopy approach across multiple centers with various operators. Many younger operators began with conventional approach, but gradually switched to zero-fluoroscopy approach. In our previous study, the zero-fluoroscopy approach during the ablation of idiopathic ventricular arrhythmias showed no obvious learning curve whereas the ablation of SVT using zero-fluoroscopy approach showed a learning curve [25]; however, the average procedure time of the ZF approach was only about 15 min longer than that of conventional approach and substantially shortened after approximately 60 cases.

Ensite NavX system can be used for accurate navigation. The advantages of three dimensional mapping system has been widely discussed in previous studies [8, 12, 25–29]. According to our experience, the guidewires must be withdrawn before model construction using Ensite NavX system for guidance since the guidewire will lead to the distortion of the impedance based field. Additionally, there are some issues that should be addressed just prior to power delivery at a high risk area, such as modifying the slow pathway during the ablation of AVNRT. Firstly, respiratory validation and recalibration by a technician should be repeated, and the exact location of the His potential should be routinely re-confirmed. Secondly, the patient should avoid large respiratory excursions and should avoid significant movement. Thirdly, the surface patch used for a system reference should be placed in an inter-scapular area, particularly in obese patients with significant abdominal movement during respiration.

In this study, only seven cases (0.7%) finally switched into conventional fluoroscopy approach as fluoroscopic angiography was required. The most common reason for the switch was severe tortuous vessels; those were usually in elderly patients over seventies. Actually, zero-fluoroscopy approach still can be succeed by advancing a long sheath if a stiff long guidewire can pass through the tortuous vessel. The less common reason was pathways suspected to be originated from a coronary sinus or a diverticulum; most of the patients showed an initial negative delta wave on lead II in surface ECG electrocardiogram; although one of them eventually were not inside the coronary sinus but were located at the superior margin of coronary sinus orifice.

We also found that noviciate was more rapidly familiar with zero-fluoroscopy navigation than experienced operators. Hence, we believe that the establishing of the method, habit, and systemic training are critical factors for familiar applying zero-fluoroscopy technique.

Our study has several limitations. First, our study was not a randomized study. Second, we did not compare other navigation systems (e.g. Carto system) versus the conventional fluoroscopy approach in this study. Finally, we acknowledge that using the Ensite NavX system adds certain procedural costs.

## Conclusions

Zero-fluoroscopy ablation of supraventricular tachycardia guided by a three dimensional electroanatomic system is as effective and safe as a conventional fluoroscopy approach with comparable procedure duration.

Further research is needed to establish the techniques and applicability of a zero-fluoroscopy approach for more complex arrhythmias. Meanwhile, continued technological advances may help realize the dream of a completely zero-fluoroscopy approach for arrhythmias ablation procedures.

## Data Availability

The datasets used and analyzed during the current study are available from the corresponding author on reasonable request.

## Abbreviations

AF: Atrial flutter
ALARA: As low as reasonably achievable
AP: Accessory pathway
AT: Atrial tachycardia
AVB: Atrial ventricular block
AVNRT: Atrioventricular nodal reentry tachycardia
AVRT: Atrioventricular reciprocating tachycardia
CA: Catheter ablation
CF: Conventional fluoroscopy
CS: Coronary sinus
EPS: Electrophysiological study
ICE: Intracardiac echocardiography
LAO: Left anterior oblique view
RA: Right atrium
RAO: Right anterior oblique view
RFCA: Radiofrequency catheter ablation
SVT: Supraventricular tachycardia
3D: Three-dimensional
TEEPS: Transesophageal electrophysiological study
ZF: Zero-fluoroscopy
